# Virotherapy combined with anti-PD-1 transiently reshapes the tumor immune environment and induces anti-tumor immunity in a preclinical PDAC model

**DOI:** 10.3389/fimmu.2022.1096162

**Published:** 2023-01-16

**Authors:** Rūta Veinalde, Gemma Pidelaserra-Martí, Coline Moulin, Chin Leng Tan, Theresa E. Schäfer, Na Kang, Claudia R. Ball, Jonas Leichsenring, Albrecht Stenzinger, Lars Kaderali, Dirk Jäger, Guy Ungerechts, Christine E. Engeland

**Affiliations:** ^1^ Clinical Cooperation Unit Virotherapy, German Cancer Research Center (DKFZ), Heidelberg, Germany; ^2^ Faculty of Health, School of Medicine, Center for Biomedical Research and Education (ZBAF), Institute of Virology and Microbiology, Witten/Herdecke University, Witten, Germany; ^3^ Faculty of Biosciences, Heidelberg University, Heidelberg, Germany; ^4^ Ecole Normale Supérieure de Lyon, Lyon, France; ^5^ Clinical Cooperation Unit Neuroimmunology and Brain Tumor Immunology, German Cancer Research Center (DKFZ), Heidelberg, Germany; ^6^ Department of Translational Medical Oncology, National Center for Tumor Diseases (NCT) and German Cancer Research Center (DKFZ), Heidelberg, Germany; ^7^ Department of Translational Medical Oncology, National Center for Tumor Diseases Dresden (NCT) Heidelberg and German Cancer Research Center (DKFZ), Heidelberg, Germany; ^8^ Center for Personalized Oncology, National Center for Tumor Diseases (NCT) Dresden and University Hospital Carl Gustav Carus, Faculty of Medicine and Technische Universität Dresden, Dresden, Germany; ^9^ Institute of Pathology, Heidelberg University Hospital, Heidelberg, Germany; ^10^ Institut für Pathologie, Zytologie und molekulare Diagnostik, Regiomed Klinikum Coburg, Coburg, Germany; ^11^ Institute for Bioinformatics, University Medicine Greifswald, Greifswald, Germany; ^12^ Clinical Cooperation Unit Applied Tumor Immunity, German Cancer Research Center (DKFZ), Heidelberg, Germany; ^13^ Department of Medical Oncology, University Hospital Heidelberg, Heidelberg, Germany and National Center for Tumor Diseases (NCT) Heidelberg, Heidelberg, Germany

**Keywords:** cancer immunotherapy, immune checkpoint, PD-1, oncolytic virus, measles vaccine, PDAC

## Abstract

**Introduction:**

Pancreatic ductal adenocarcinoma (PDAC) is largely refractory to cancer immunotherapy with PD-1 immune checkpoint blockade (ICB). Oncolytic virotherapy has been shown to synergize with ICB. In this work, we investigated the combination of anti-PD-1 and oncolytic measles vaccine in an immunocompetent transplantable PDAC mouse model.

**Methods:**

We characterized tumor-infiltrating T cells by immunohistochemistry, flow cytometry and T cell receptor sequencing. Further, we performed gene expression profiling of tumor samples at baseline, after treatment, and when tumors progressed. Moreover, we analyzed systemic anti-tumor and anti-viral immunity.

**Results:**

Combination treatment significantly prolonged survival compared to monotherapies. Tumor-infiltrating immune cells were increased after virotherapy. Gene expression profiling revealed a unique, but transient signature of immune activation after combination treatment. However, systemic anti-tumor immunity was induced by virotherapy and remained detectable even when tumors progressed. Anti-PD-1 treatment did not impact anti-viral immunity.

**Discussion:**

Our results indicate that combined virotherapy and ICB induces anti-tumor immunity and reshapes the tumor immune environment. However, further refinement of this approach may be required to develop its full potential and achieve durable efficacy.

## 1 Introduction

The unprecedented success of immune checkpoint blockade (ICB) targeting programmed cell death protein 1 (PD-1) and its ligand PD-L1 marked the renaissance of cancer immunotherapy (1). However, a large proportion of patients, especially also those afflicted by poorly immunogenic tumors such as pancreatic ductal adenocarcinoma (PDAC), do not benefit from ICB monotherapy (2). Therefore, combination treatments are sought that render resistant tumors responsive. Possible reasons for lack of response include low tumor antigenicity, absence of intratumoral T cells, and an immunosuppressive microenvironment. Many preclinical and several clinical studies have demonstrated that oncolytic virotherapy represents a promising strategy to overcome these limitations. Viral oncolysis results in the release of tumor antigens in a highly immunostimulatory context, leading to immune cell influx and reshaping of the tumor immune environment (3). Several oncolytic virus therapeutics are currently in clinical development (4). Among these, measles vaccine strains (MV) stand out in terms of safety and broad tumor tropism (5).

We and others have previously reported efficacy of MV and ICB combination therapy in immunocompetent mouse models of melanoma, colorectal cancer, and glioblastoma (6–10). These studies showed delayed tumor progression and prolonged survival, an increased intratumoral T cell infiltration, an increased ratio of effector to regulatory T cells, and enhancement of tumor-specific T cell responses upon MV and ICB combination treatment.

In the present study, we intended to gain further insights into efficacy and mechanisms of action of combined oncolytic measles virotherapy and anti-PD-1 immune checkpoint blockade. We focused on PDAC as a target tumor entity with high medical need and primary resistance to ICB (11). We employed MV-NIS, an oncolytic agent currently investigated in clinical trials (4, 12) (clinicaltrials.gov NCT03171493, NCT02962167, NCT02068794, NCT02364713, NCT02700230). MV-NIS has also been employed in a preclinical study with PDAC xenografts in combination with ^131^I radiotherapy (13). Our results indicate synergistic effects of MV and ICB combination therapy in PDAC. We detect induction of systemic anti-tumor immunity and remodeling of the local tumor microenvironment. However, the local effects are transient, demonstrating the need for further development of this approach to achieve durable efficacy.

## 2 Materials and methods

### 2.1 Cell lines

The African green monkey kidney cell line Vero was obtained from the American Type Culture Collection (CCL-81). FC1245 cells, derived from the KPC model (14), were obtained from A. Nowrouzi (DKFZ Heidelberg). FC1245-CD46 expressing the MV entry receptor human CD46 were generated by lentiviral transduction as described previously (15). B16-CD46 and MC38-CD46 were generated by stable transfection. Vero and MC38-CD46 cells were cultivated in Dulbecco’s modified Eagle’s medium (DMEM, Life Technologies) supplemented with 10% fetal calf serum (FCS, Biosera). B16-CD46, FC1245, and FC1245-CD46 cells were cultivated in Roswell Park Memorial Institute (RPMI, Life Technologies) medium with 10% FCS. All cell lines were cultivated at 37^o^C in a humidified atmosphere with 5% CO_2_. Routine tests for mycoplasma contamination were performed.

### 2.2 Patient-derived pancreatic cancer cultures

All experiments with human material were performed in accordance with the Declaration of Helsinki and were approved by the ethics committee of the Medical Faculty of Heidelberg University (323/2004, Amendment 03). Informed consent was received from participants before study inclusion. Generation and cultivation of patient-derived PDAC cultures have been described previously (16). Cultures were subjected to SNP typing and Multiplex Cell Contamination Testing (Multiplexion). Cultures were grown in DMEM Advanced F12 medium supplemented with 0.6% (w/v) glucose, 2 mM L-glutamine, 2% B27 supplement (1×) (all Thermo Fisher Scientific), 12 μg/ml heparin and 5 mM HEPES buffer (both Sigma Aldrich), 10 ng/ml rhFGF basic, 20 ng/ml rhFGF-10, and 20 ng/ml rhNodal (all R&D Systems). Cytokines were renewed twice per week.

### 2.3 Oncolytic measles virus

MV-NIS is derived from the Edmonston vaccine strain of measles virus and encodes the sodium iodide symporter (NIS) (17). High-titer purified MV-NIS was obtained from Imanis Life Sciences. Measles Schwarz vaccine strain (MV) and its derivate encoding eGFP were generated and propagated as described previously (8, 18). Procedures for titration and characterization of recombinant measles vectors were carried out as in (18). To ensure comparability, all titrations were performed with Vero cells.

### 2.4 Virus infection and LDH assay

For infection, patient-derived PDAC cultures were seeded at a density of 1×10^3^ cells per well in 12 well plates and inoculated with MV-NIS at a multiplicity of infection (MOI) of 0.03 and 3 in triplicates, respectively. The inoculum was replaced with fresh medium 2 h post infection. Cell lysis was determined by lactate dehydrogenase (LDH) release assay. For 100% LDH release samples, mock infected cells were harvested 24 h post inoculation, subjected to two freeze-thaw cycles for complete cell lysis, and stored at -80°C. Culture supernatants were collected at designated time points, centrifuged (380 × g, 5 min) and stored at -80°C. LDH release into the supernatants was quantified using the CytoTox 96 Non Radioactive Cytotoxicity Assay kit (Promega). Relative cytotoxicity was calculated by normalization to 100% LDH release controls.

Murine tumor cells were seeded at a density of 1×10^5^ cells per well in 12 well plates and inoculated with MV-NIS at an MOI of 3 or with a measles vaccine derivative encoding eGFP at an MOI of 1. The inoculum was replaced with fresh medium 2 h post infection.

For *ex vivo* infections of FC1245-CD46, tumors were explanted from euthanized mice. Tumors were minced with a scalpel, passed through a 100 µm filter and cultivated in RPMI + 10% FCS with 1% antibiotic-antimycotic solution (Sigma) until confluent. *Ex vivo* cultures were inoculated with the measles vaccine variant encoding eGFP at an MOI of 1 as described above.

### 2.5 Animal experiments

All experimental procedures with animals were approved by the regional council (Regierungspräsidium Karlsruhe, protocols G-192/15, G-58/17, G-17/19) and were performed in compliance with the German Animal Protection Law and the institutional guidelines. Six- to eight-week old female C57BL/6J mice were acquired from Harlan Laboratories. Mice were housed in groups of five in individually ventilated cages under specific pathogen-free (SPF) conditions at the Center for Preclinical Research of the German Cancer Research Center. After one week of acclimatization, 1x10^6^ FC1245-CD46 cells in 100 µl PBS were injected subcutaneously into the flanks of mice. For efficacy experiments, mice were assigned to treatment groups (n = 10 per group) when tumors reached an average size of 75 mm³, ensuring equal distribution of tumor sizes. Group size was calculated using nquery advisor 6.01 to enable detection of an effect with P ≈ 75% on tumor growth with a power of 80%, where P is defined as the probability that one subject in one group has a higher or lower value than a subject in the control group. Treatment was initiated as depicted in the schematics. Investigators were blinded to group assignment. MV-NIS (1x10^6^ ciu in 100 µl PBS) or 100 µl PBS (controls) were injected intratumorally on four consecutive days. Anti-PD-1 (clone J43, eBioscience; 100 µg in 200 µl PBS) or 200 µl PBS (controls) was injected intraperitoneally on the third day of virus treatment and every third day thereafter for a total of four doses. Tumor volume for each individual was monitored three times per week and was calculated using the formula


$$largest\;diameter\times smallest\;{diameter}^{2}\times 0.5$$

Animals were sacrificed when one of the following pre-defined endpoint criteria was reached: tumor volume exceeded 1000 mm^3^, the largest tumor diameter exceeded 15 mm, or tumor ulceration occurred. To minimize potential confounders, the order of treatments and measurements was varied. No animals were excluded from the efficacy analysis.

### 2.6 Immunohistochemistry

Paraffin sections of tumor tissue were prepared for immunohistochemical staining of PD-L1 and CD3. Deparaffinization and tissue staining were performed using a Benchmark Ultra IHC Staining module according to standard protocols (Ventana PD-L1 assay, clone SP263 and CONFIRM anti-CD3 Primary Antibody, clone 2GV6; Roche). Hematoxylin/eosin was used for counterstaining. IHC stainings were evaluated by a specialist in pathology according to standardized criteria (19).

### 2.7 Flow cytometry

To assess surface expression of human CD46, murine tumor cells were stained with 1 μl anti-human CD46 APC (clone TRA-2-10, Biolegend) in 100 μl PBS for 30 min on ice in the dark. Viability was assessed by staining with 0.2 μg/ml DAPI. Samples were acquired on a CytoFLEX flow cytometer (Beckman Coulter) and data were analyzed with FlowJo software (version 10.8.1, Tree Star Inc.).

To assess PD-L1 expression after measles virus infection, cells were infected at MOI 3 in 12 well plates and harvested 24 h and 48 h post infection. Patient-derived cells were stained with 1 µl anti-human PD-L1 PE (clone 29E.2A3, Biolegend) and murine cells were stained with 1 µl anti-mouse PD-L1 PE (clone 10F.9G2, Biolegend) in 100 µl PBS for 30 min at room temperature in the dark and with 0.2 μg/ml DAPI.

Samples of tumors and tumor-draining lymph nodes were prepared for flow cytometry as described in (20). In brief, fresh samples were minced, treated with 200 U/ml Collagenase I (Thermo Fisher Scientific) and passed through 100 µm cell strainers. 2x10^6^ cells in 100 µl PBS were used for staining. Prior to staining, samples were incubated with anti-mouse CD16/CD32 (Mouse Fc Block, BD Biosciences).

Tumor samples were stained with the following antibodies: 1 µl anti-CD45.2 PE/Cy7 (clone 104, Biolegend), 1 µl anti-CD3 PerCP-Cy5.5 (clone 17A2, BD Biosciences), 1 µl anti-CD4 APC-Cy7 (clone GK1.5, BD Biosciences), 1 µl anti-CD8a APC (clone 53-6.7, BD Biosciences), 1 µl anti-CD335 (NKp46) FITC (clone 29A1.4, Biolegend), 1 µl anti-CD69 PE (clone H1.2F3, Biolegend). Tumor-draining lymph node samples were stained with the following antibodies: 1 µl anti-CD3 PerCP-Cy5.5 (clone 17A2), 1 µl anti-CD4 APC-Cy7 (clone GK1.5), 1 µl anti-CD8a APC (clone 53-6.7) (all from BD Biosciences), 1 µl anti-CD44 PE (clone IM7), 1 µl anti-CD62L FITC (clone MEL-14) (both from Biolegend). After antibody staining for 30 min in the dark, washing, and staining with 0.2 µg/ml DAPI, acquisition was performed on a BD FACS LSR II (BD Biosciences). Data were analyzed using FlowJo software (version 10.0.7r2, Tree Star Inc.).

### 2.8 T cell receptor profiling

DNA was extracted from tumor samples using the DNeasy Blood and Tissue Kit (Qiagen). TCRB survey sequencing was performed by Adaptive Biotechnologies. Data were analyzed using vdjtools (21). Gini index was calculated using only productive TCRB with ineq (v0.2-13) package in R and plotted with ggplot2 (v3.3.5). Morisita index and heatmap were calculated and visualized using immunarch (v0.6.7) package in R (v4.0.5).

### 2.9 Gene expression profiling

Total RNA was extracted from tumor samples using the RNeasy Mini Kit (Qiagen). NanoString gene expression analysis was performed by the nCounter Core Facility Heidelberg. In short, 25 ng RNA from total RNA samples was subjected to quality control by Agilent Bioanalyzer 2100 and Qubit system, hybridized with the CodeSet Immunology Panel (Mouse; NanoString Technologies), and processed using nCounter SPRINT Profiler. Raw data were normalized to the set of internal reference genes included in the CodeSet panel and data were analyzed using nSolver 4.0 software including the Advanced Analysis package (NanoString Technologies) for cell profiling and pathway analysis. For pathway analysis, genes differentially expressed between mock and treatment groups (p *<* 0.05) were mapped onto pathways using nSolver and log2-fold change expression values were calculated for each gene. Differential gene expression analysis was performed in R version 3.6.0 with the Bioconductor package “NanoStringDiff” (22). Data were normalized based on positive controls and housekeeping genes and corrected for background. A generalized linear model (GLM) likelihood ratio test was used as statistical test. Details are described in (22).

### 2.10 qRT-PCR

One microgram of tumor RNA extracted for gene expression profiling was reverse transcribed using Maxima H Minus RT (Thermo Fisher Scientific) with Olidgo(dT) primers. Quantitative PCR was conducted on a CFX96 Real-Time System (BioRad) with 1 μl cDNA or standard, 0.13 μl forward primer at 33 μM, 0.13 μl reverse primer at 33 μM, and 10.5 μl Power SYBR Green PCR Master Mix (Thermo Fisher Scientific) in a total volume of 20 µl. Standards consisted of 10-fold serial dilutions of a plasmid encoding MV N, pCG N (23), starting with 1x10^7^ gene copies/μl. The following primers were used:

N-241 (5’ – TTACCACTCGATCCAGACTTC – 3’)
N-331+ (5’ – CCTATTAGTGCCCCTGTTAGTTT – 3’)

The data were analyzed using CFX Manager Software (version 3.1, BioRad).

### 2.11 Interferon-γ ELISpot

FC1245-CD46 tumors were established in six- to eight-week old female C57BL/6J mice (Janvier) and treated as described above. However, since MV-NIS was no longer commercially available, unmodified measles Schwarz vaccine (8), 1x10^6^ ciu in 100 µl OptiMEM per dose) or 100 µl OptiMEM (mock and anti-PD-1 groups) were used for treatment. Anti-PD-1 (clone J43) was obtained from BioXCell. Mice were sacrificed for spleen extraction at t1 (day 7 after treatment initiation) and t2 (day 13 after treatment initiation). Spleens were passed through 100 μm cell strainers, subjected to erythrocyte lysis with ACK Lysing Buffer (Thermo Fisher Scientific) for 10 min at room temperature and cultured in RPMI + 10% FCS + 1% penicillin/streptomycin.

To assess anti-tumor immunity, 1x10^6^ splenocytes were cultured with 1x10^5^ FC1245-CD46 target cells at an effector to target ratio of 10:1. To analyze measles-specific immunity, 5x10^5^ splenocytes were cultured with 7 μg/ml measles virus premium bulk antigen (Serion Immunologics, t1) or incubated with MV at an MOI of 0.5 (t2). Incubations with 10 μg/ml Concanavalin A (ConA, Sigma-Aldrich) or medium served as positive and negative controls, respectively. ELISpot co-cultures were established in clear 96-well MultiScreen_HTS_-IP filter plates with a 0.45 μm pore size hydrophobic PVDF membrane (Merck) in a total volume of 200 µl. ELISpot was conducted using the mouse IFN-γ ELISpot pair comprising capture and detection antibodies (RRID: AB_2868948, BD Biosciences). Plates were coated overnight at 4°C with mouse IFN-γ ELISpot capture antibody prior to co-culture set-up. After 40 h of co-culture, ELISpots were developed using mouse IFN-γ ELISpot detection antibody, Streptavidin-HRP (BD Biosciences), and TMB substrate (Mabtech) according to manufacturer’s instructions. Data were acquired on a Bioreader 7000-E instrument (Biosys) and analyzed using EazyReader® software. Saturated wells were set to 450 spots. Splenocyte samples with an average spot count below 250 in the positive control (ConA) were excluded from analysis. To test for CD46 dependency of anti-tumor immune responses, 1x10^6^ splenocytes were cultured with 1x10^5^ FC1245 or FC1245-CD46 target cells at an effector to target ratio of 10:1. ELISpots were developed as described above.

### 2.12 Statistics

Statistical analyses and data visualization were performed using Graph Pad Prism software (Versions 8.4.3 and 9.2.0, GraphPad Software, LLC). Comparisons of multiple groups were performed using ANOVA with Tukey’s post-test as indicated. Survival data were analyzed by Mantel-Cox (log rank) test with Bonferroni correction for multiple comparisons.

## 3 Results

### 3.1 MV-NIS treatment and PD-L1 expression of patient-derived PDAC cultures

**Figure 1:**
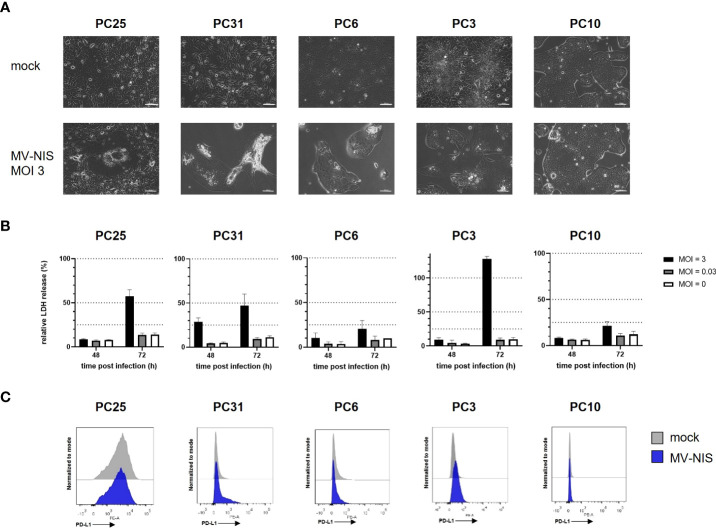
MV-NIS treatment and PD-L1 expression of patient-derived PDAC cultures. Patient-derived PDAC cultures were infected with MV-NIS at a multiplicity of infection (MOI) of 3 or subjected to mock infection. **(A)** Microscopic images were acquired 48 hours post infection (h p.i.). Scale bars indicate 100 µm. **(B)** MV-induced cytolysis was assessed by LDH release assay at 48 and 72 h p.i. Relative cytotoxicity was calculated using a 100% lysis control for each culture. Mean values and standard deviation from three replicates are shown. **(C)** Fourty-eight h p.i., cells were stained with a PE-labeled PD-L1 specific antibody and analyzed by flow cytometry. Histograms depicting PE fluorescence intensity are shown. Representative plots from n = 3 independent experiments are shown.

Patient-derived PDAC cultures which recapitulate hallmarks of human PDAC (16) were treated with MV-NIS or left untreated. Morphological differences were observed between cultures, signifying intertumoral heterogeneity. For instance, PC10 grew in islets while PC25 grew as a monolayer. Treatment with MV-NIS led to cytopathic effects, which differed between cultures. Syncytia formation, a hallmark of measles virus infection, was visible in PC25. Disruption of the cell monolayer was observed in PC31 and PC6. In other cultures, such as PC10, no major differences in culture morphology were observed upon infection with MV-NIS compared to mock treatment (**Figure 1A**). Direct oncolytic effects as determined by LDH release assay were also variable between cultures from different individuals (**Figure 1B**), reflecting tumor heterogeneity. Expression of the MV receptor CD46 did not correlate with oncolytic efficacy (Schäfer et al., unpublished data). Moreover, MV replication as determined by titration of viral progeny was modest in patient-derived PDAC cultures (**Supplementary Figure S1**) compared to most human pancreatic cancer cell lines (24, 25). Thus, direct oncolytic activity of MV is most likely limited in PDAC. However, PDAC may be amenable to MV-mediated immunotherapy. Treatment with MV-NIS led to upregulation of PD-L1 on some cultures (**Figure 1C**), which is in line with previous reports (6, 7, 10). However, baseline expression and degree of PD-L1 upregulation were also variable. There was no direct correlation between MV permissiveness and PD-L1 upregulation. Overall, PD-L1 upregulation upon MV-NIS treatment provided a rationale for testing the combination of MV-NIS and PD-1/PD-L1 checkpoint blockade in PDAC.

### 3.2 MV-NIS combined with PD-1 checkpoint blockade in murine PDAC

We intended to further study the combination of MV and PD-1 checkpoint blockade in an immunocompentent murine PDAC model. Therefore, we tested viral replication, direct oncolytic effects and PD-L1 expression after treatment of FC1245-CD46 cells with MV-NIS. These cells are derived from the KPC mouse model (14) and stably express the MV entry receptor CD46 at levels comparable to other engineered murine cell lines (**Figure S2A**). Similar to other murine cells, FC1245-CD46 shows limited MV replication and limited sensitivity to direct MV oncolysis *in vitro* and *ex vivo* (**Supplementary Figures S2B–F**). As patient-derived PDAC cultures also showed modest viral replication and oncolysis upon treatment with MV-NIS (**Figures S1**, **1B**), we deemed FC1245-CD46 an appropriate model to study MV-mediated immunotherapy. Murine tumor cells cultured *in vitro* showed low expression levels of PD-L1, which were not or only slightly upregulated after inoculation with MV-NIS. Interestingly, treatment with the measles virus Schwarz vaccine strain led to stronger induction of PD-L1 than MV-NIS (**Supplementary Figure S2G**).

**Figure 2:**
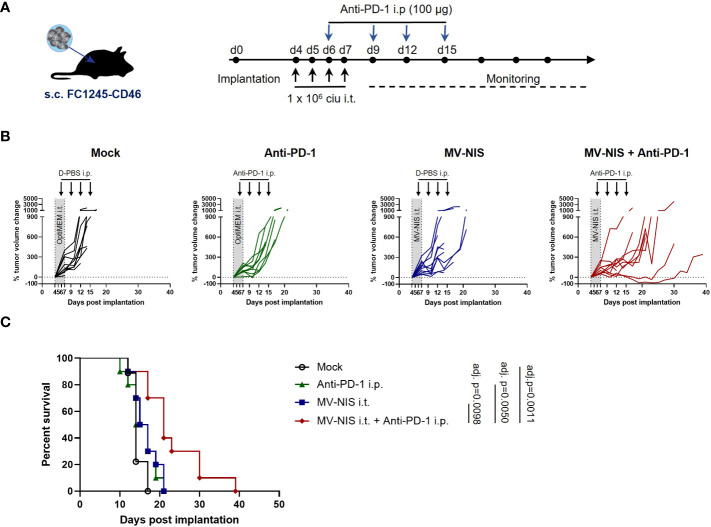
Efficacy of MV-NIS and anti-PD-1 in an immunocompetent PDAC model. **(A)** Treatment schedule. FC1245-CD46 cells were implanted subcutaneously into the flanks of C57BL/6 mice. When tumors reached an average volume of 75 mm³ (day 4 post implantation), treatment was initiated according to the depicted schedule. Animals received intratumoral injections of 1x10^6^ cell infectious units (ciu) of MV-NIS or PBS (control groups) on four consecutive days. From day 3 of virus treatment onwards, animals received intraperitoneal injections of 100 µg of anti-PD-1 antibody or PBS (control groups) every third day for a total of four doses. **(B)** Individual tumor growth curves for mice treated with PBS only (mock), MV-NIS only, anti-PD-1 only or the combination of MV-NIS and anti-PD-1. Relative changes in tumor volume in comparison to pre-treatment tumor volume over time are shown. **(C)** Kaplan-Meier survival analysis. Data were analyzed using Mantel-Cox (log rank) test with Bonferroni correction for multiple comparisons.

To test efficacy of MV combined with PD-1 checkpoint blockade, C57BL/6J mice bearing subcutaneous FC1245-CD46 tumors were subjected to mock treatment, treatment with intratumoral (i.t.) injections of MV-NIS, intraperitoneal (i.p.) injections of anti-PD-1, or the combination of i.t. MV-NIS and i.p. anti-PD-1, as outlined in **Figure 2A**. In this very aggressive model, mock treated animals showed rapid tumor progression (**Figure 2B**), reaching endpoint criteria within 12 to 17 days (median 14 days) after tumor implantation (**Figure 2C**). Monotherapy with either MV-NIS or anti-PD-1 had only limited effects on tumor growth and outcome, with a median time to endpoint of 16 days (**Figures 2B, C**). In contrast, combination treatment with MV-NIS and anti-PD-1 delayed tumor progression and prolonged survival significantly (**Figures 2B, C**). Nevertheless, all tumors ultimately progressed, reaching endpoint criteria at a median of 21 days. The benefits of combined MV-NIS plus anti-PD-1 compared to monotherapies were reproducible in an independent experiment (**Supplementary Figure S3**).

### 3.3 Immune analysis after MV-NIS and anti-PD-1 treatment

**Figure 3:**
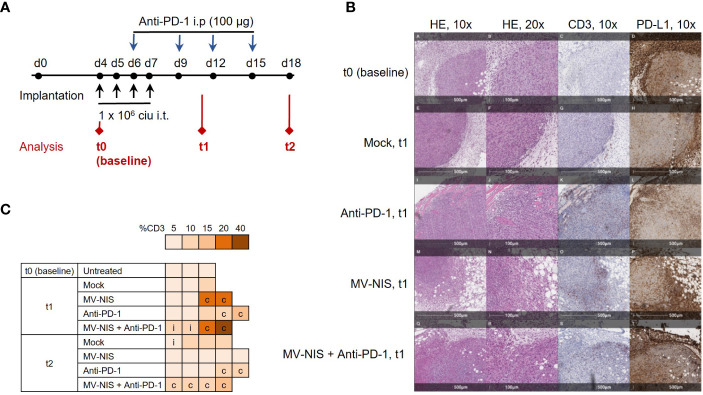
Tumor immune environment after MV-NIS and anti-PD-1 treatment. **(A)** Experimental outline. C57BL/6 mice bearing subcutaneous FC1245-CD46 tumors were treated as described above. Tumor samples for immuno-analyses were collected at depicted timepoints t0 (baseline), t1, and t2. **(B)** Histology and immunohistochemistry. Tumor sections collected at t0, t1, and t2 were stained with hematoxylin/eosin (HE) and with antibodies specific for CD3 and PD-L1. Representative sections are shown. Scale bars correspond to 500 µm or 100 µm, respectively (as indicated). **(C)** CD3 expression. CD3 staining was used to determine the percentage of tumor stroma containing mononuclear immune cells according to (19). The percentage of CD3+ tissue is indicated by the color code; c indicates circumferential, i indicates intratumoral immune infiltration.

To gain insights into mechanisms underlying efficacy of MV-NIS and anti-PD-1 combination treatment, mice bearing subcutaneous FC1245-CD46 tumors were treated as described above and tumor samples were collected as depicted in **Figure 3A**, with timepoints at baseline (t0, before treatment), one week (t1) and two weeks (t2) after treatment initiation. In line with our *in vitro* data, qRT-PCR of tumor samples indicated limited viral replication, with Cq values just above the detection limit at t1 after MV-NIS or combination treatment (**Figure S4**). This indicates that direct oncolysis does not contribute substantially to anti-tumor efficacy. Since intratumoral T cell infiltration has been identified as an indicator of response to immunotherapy (26) and prognosis (27, 28) in PDAC, the quantity and distribution of CD3+ cells in tumor sections were assessed (**Figures 3B, C** and **Supplementary Table 1**). Histopathological evaluation indicated increased CD3+ T cell infiltration in individual tumors after MV-NIS and MV-NIS plus anti-PD-1 combination treatment, but not after anti-PD-1 monotherapy, compared to mock treatment at t1. Slight increases compared to mock treatment were also observed for MV-NIS plus anti-PD-1 combination treatment at t2. The location of the CD3+ T cell infiltrate was circumferential in most samples.

As another potential marker for response to immunotherapy (26), PD-L1 expression within the tumor and tumor stroma was also assessed (**Figure 3B** and **Supplementary Table 1**). Over 90% of stromal cells stained strongly positive for PD-L1 in all tumor sections from all treatment groups. Interestingly, tumor cell PD-L1 expression decreased from over 90% at baseline and t1 to 70% and lower at t2 in all treatment groups.

**Figure 4:**
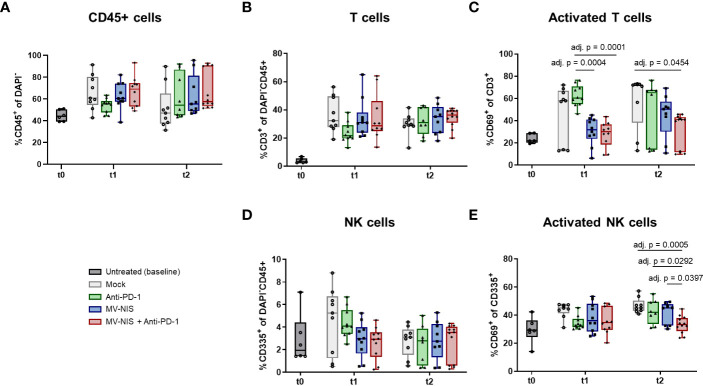
Tumor-infiltrating lymphocytes after MV-NIS and anti-PD-1 treatment. After treatment as depicted in **Figure 3A**, tumors were explanted at t0, t1, and t2. Tumor samples were prepared for flow cytometry. The percentages of **(A)** CD45+ cells among live cells (DAPI-), **(B)** T cells (CD3+) among live CD45+ cells, **(C)** activated T cells (CD3+ CD69+) among live T cells, **(D)** NK cells (CD335+) among live CD45+ cells, **(E)** activated NK cells (CD335+ CD69+) among live NK cells were determined. Box and whisker plots with whiskers depicting minimal and maximal detected abundances as well as median in each group are shown. Data were analyzed by one-way ANOVA for each timepoint with Tukey’s post-test. Multiplicity-adjusted p values < 0.05 are shown. adj. p = adjusted p value.

To complement these data, tumor-infiltrating lymphocytes were assessed by flow cytometry at baseline, t1, and t2 (**Figure 4**). There was a slight, statistically non-significant increase in abundance of CD45+ cells after combination treatment at t1 compared to the other treatments (**Figure 4A**). There were no statistically significant differences in the relative abundance of CD3+ cells among all CD45+ cells (**Figure 4B**). Interestingly, MV-NIS and combination treatment led to a significant decrease in the relative abundance of CD3+ cells expressing the early activation marker CD69 compared to anti-PD-1 at t1 and mock at t2 (**Figure 4C**). Since natural killer (NK) cells have also been implicated in response to PD-1/PD-L1 checkpoint blockade (29), the relative abundance of CD335+ and activated CD335+ CD69+ NK cells was also determined (**Figures 4D, E**). The abundance of NK cells varied considerably between tumors and was not significantly different between the treatment groups at t1 and t2. Interestingly, these analyses yielded a significantly decreased abundance of NK cells expressing CD69 in the combination therapy compared to the monotherapies and mock treatment at t2.

Further, we analyzed T cells in tumor-draining lymph nodes (TDLN) by flow cytometry (**Supplementary Figures S5**, **S6**). While there were no significant differences in total CD3+ T cells in TDLN (**Supplementary Figure S6A**), there was a significant increase in the effector memory CD4+ population after MV-NIS monotherapy or combination treatment compared to anti-PD-1 monotherapy at t1 (**Supplementary Figure S6E**). Moreover, the central memory CD4+ population was significantly more abundant in TDLNs from mice treated with MV-NIS as compared to anti-PD-1 or mock at t1 (**Supplementary Figure S6F**). At t2, central memory CD4+ and CD8+ T cells were significantly increased in the combination group compared to the MV-NIS group (**Supplementary Figures S6F, I**).

**Figure 5:**
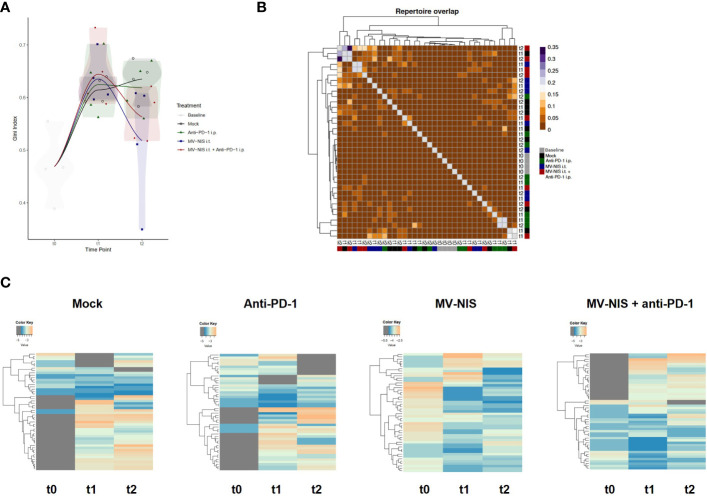
T cell receptor repertoire of tumor-infiltrating T cells. DNA was extracted from FC1245-CD46 tumor samples collected at indicated timepoints and used for TCRβ sequencing. **(A)** Gini index. **(B)** Morisita’s Horn overlap index. **(C)** Clonotype tracking was performed with pooled data from four mice in each group at each timepoint based on strict intersection rule using vdjtools. Each row represents one clone, with grey indicating absence of a clone at the respective timepoint.

We reasoned that not the quantity, but rather the quality of tumor-infiltrating lymphocytes may determine response to immunovirotherapy. Therefore, we performed TCR sequencing with tumor samples collected at baseline, t1, and t2. There were no overt differences in V and J segment usage and quantile statistics between the treatment groups (**Supplementary Figure S7**). To further analyze clonality, the Gini index was calculated (**Figure 5A**), where 0 corresponds to minimal clonality and maximal diversity (that is, several clonotypes all having the same frequency), and 1 corresponds to the highest clonality (that is, dominance of few clonotypes). The Gini index was higher in all groups at t1 and t2 as compared to baseline (t0). No overt differences we observed in clonality of the intratumoral T cell repertoire between the different groups. Morisita**’**s Horn overlap index (**Figure 5B**) indicated a higher overlap in the TCR repertoire in samples from the MV-NIS and combination treatment groups, with some variability between different samples. In line with this, clonal tracking (**Figure 5C**) showed an overlap between clones from t1 and t2 in the MV-NIS and MV-NIS plus anti-PD-1 combination treatment groups, which might represent virus-reactive or tumor-reactive T cells.

**Figure 6:**
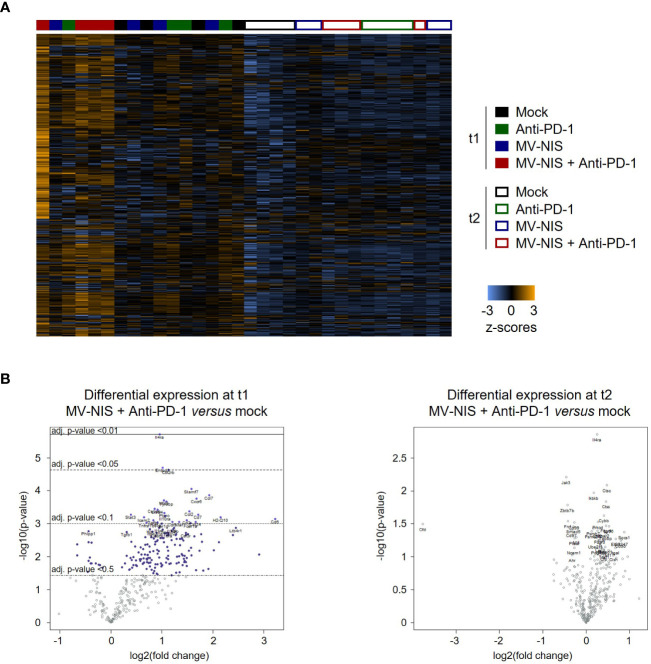
Gene expression profiling. **(A)** Dendrogram and heatmap depicting gene expression profiles of n = 3-4 samples for each timepoint and treatment. Analysis was performed with the Advanced Analysis package of nSolver 4.0 using hierarchical clustering. **(B)** Volcano plots showing differentially expressed genes between mock and MV-NIS plus anti-PD-1 treatment at t1 and t2. Analyses were performed with the Advanced Analysis package from nSolver 4.0.

We performed targeted transcriptome analysis using the Nanostring nCounter mouse immunology panel to gain insights into alternative immune-mediated mechanisms underlying initial response and subsequent tumor progression upon MV-NIS and anti-PD-1 combination treatment. Nanostring gene expression profiles revealed a unique expression pattern in mice treated with MV-NIS plus anti-PD-1 at t1, which was lost at t2 (**Figure 6**). Immune pathway analysis using nSolver software (**Supplementary Figure S8**) pointed towards enhanced adaptive immunity, cytokine secretion, chemokine signaling, cell adhesion, MHC I antigen presentation, T cell receptor signaling, and lymphocyte activation at t1 after combination treatment with MV-NIS and anti-PD-1 compared to the other treatment groups. Of note, also innate immune activation, genes associated with host-pathogen interaction and innate immune signaling were moderately elevated after combination treatment at t1, but not after monotherapy. These differences in gene expression signatures were no longer observed at t2.

Immune cell deconvolution analysis with nSolver (**Supplementary Figure S9**) indicated an increased abundance of total CD45+ cells at t1 after MV-NIS and anti-PD-1 combination treatment. T cells, cytotoxic cells, and dendritic cells (DCs) appeared to be increased, and also macrophages as well as neutrophils. Interestingly, the gene signature associated with exhausted CD8+ T cells was also increased. After treatment with anti-PD-1 monotherapy moderate, but non-significant increases in DC- and neutrophil-associated genes were detected. No enrichment of specific immune cells was detected for MV-NIS monotherapy at this timepoint. Remarkably, at t2, the abundance of total CD45+ cells and cytotoxic cells appeared to be increased in this group, whereas the effects seen at t1 in the combination group had been lost. At large, the immune cell deconvolution analysis was consistent with the flow cytometry data on tumor-infiltrating lymphocytes (compare **Figure 4**) with overall less variability in the former.

To dissect the unique immune activation profile after combination treatment at t1, we performed differential gene expression analysis with tumor samples from the different treatment groups (**Supplementary Table 2**). Compared to anti-PD-1 monotherapy, combination treatment led to increased intratumoral expression of T cell-associated genes (*Cd4*, *Cd8b*), costimulatory molecules (*Cd27*, *Cd40l*) as well as genes linked to lymphocyte activation (*Cd69*, *Cd5*, *Cd6*, *Slamf1*, *Tnfrsf8*) and cytotoxic effector cell function and differentiation as well as Th1 polarization (*Il12a*, *Il12rb2*, *Runx3* and *Tbx21*, i.e. *Tbet*).

Overall, these data indicate broad immune activation and especially activation of effector T cells at t1 after combination treatment with MV-NIS and anti-PD-1. This immune signature was unique to the combination and not observed after either monotherapy. However, effects were lost at t2 (**Supplementary Table 3**), indicating that the effects on the tumor immune environment were transient.

### 3.4 Systemic anti-PDAC immunity induced by virotherapy

**Figure 7:**
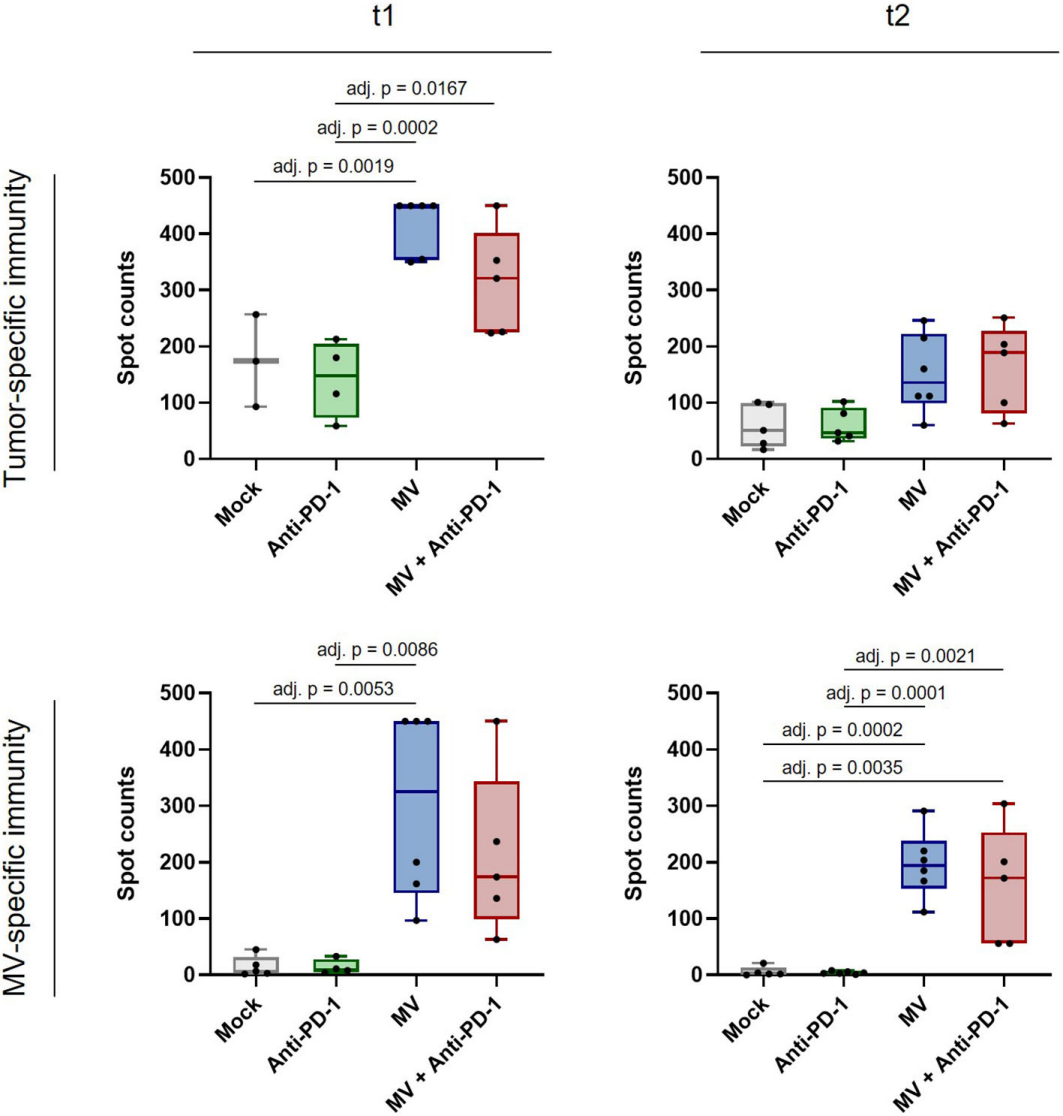
Anti-tumor and anti-measles immunity after MV and anti-PD-1 treatment. C57BL/6J mice bearing subcutaneous FC1245-CD46 tumors were treated as described in methods, with treatment starting on day 4 after tumor implantation. Spleens were collected at t1 and t2, co-cultured with indicated stimuli and IFN-γ ELISpot was conducted. Box and whisker plots with whiskers depicting minimal and maximal values as well as median in each group are shown. Dots represent individual mice. Saturated wells were set to 450 spots. Data were analyzed using one-way ANOVA with Tukey’s correction for multiple comparisons. adj. p = adjusted p value.

Finally we assessed whether MV plus anti-PD-1 induces not only local immunomodulation, but also systemic anti-tumor immunity in PDAC. We performed IFN-γ ELISpot analysis with splenocytes from mice from all treatment groups at t1 and t2 (**Figure 7**). While splenocytes from mice treated with anti-PD-1 alone did not show enhanced tumor-specific reactivity compared to mock, we found significantly increased tumor-specific IFN-γ responses after treatment with MV at t1 (**Figure 7**, top left panel, adj. p **<** 0.01 between MV and mock). Combination with anti-PD-1 yielded similar reactivity as MV alone, indicating that anti-tumor immunity is driven by virotherapy. Remarkably, despite loss of the immune activation signature in the tumor at t2, systemic anti-tumor immunity was maintained, although the differences were no longer statistically significant (**Figure 7**, top right panel). Moreover, virotherapy induced MV-specific immunity at t1, which was maintained at t2 (**Figure 7**, bottom panels, adj. p **<** 0.01 between MV and mock at t1, adj. p **<** 0.001 between MV and mock at t2). Combination with anti-PD-1 did not affect anti-MV immunity.

Further, we confirmed that anti-tumor immunity does not depend on tumor CD46 expression by stimulation of splenocytes with both parental and CD46-positive FC1245 cells (**Supplementary Figure S10**).

Overall, our results indicate that MV virotherapy can induce durable systemic anti-tumor immunity in PDAC, whereas peripheral immune signatures are transiently modulated by combination with anti-PD-1.

## 4 Discussion

In this study, we aimed at deciphering possible mechanisms of immunotherapeutic synergy of oncolytic virotherapy and PD-1 checkpoint blockade.

Preclinical efficacy of combined virotherapy and PD-1/PD-L1 checkpoint blockade has been demonstrated across a wide range of oncolytic virus platforms and tumor models (7, 30–41). So far, few studies have assessed this approach in immunocompetent PDAC models. Kanaya et al. reported that treatment with intratumoral oncolytic adenovirus combined with systemic anti-PD-1 antibody led to prolonged survival and increased CD8+ T cell infiltration in a subcutaneous PAN02 model (42). Correlative analyses in preclinical models have found CD8+ and Th1 T cell activation, increased ratios of effector to regulatory T cells, and increased intratumoral IFN-γ expression after OV and anti-PD-1 combination treatment (43). Further, this combination was shown to promote a broader spectrum of neoantigen-specific CD8+ T cell responses (30, 38). In most settings, efficacy depends on CD8+ T cells (41, 44), but NK cells and type I interferon (44) as well as macrophage polarization (34) have also been implicated in combined OV and checkpoint blockade therapy.

Oncolytic virotherapy combined with PD-1/PD-L1 immune checkpoint antibodies is of significant clinical interest. Treatment of advanced melanoma with talimogene laherparepvec (T-VEC, Imlygic), the oncolytic herpes simplex virus approved by the US Food and Drug Administration (FDA) and European Medicines Agency (EMA), achieved a response rate of 62% in combination with the anti-PD-1 antibody pembrolizumab in a Phase Ib trial (45). However, the Phase III trial of T-VEC plus pembrolizumab was terminated due to futility (clinicaltrials.gov, NCT02263508). Nevertheless, trials investigating PD-1 blockade combined with different OVs - including herpes virus, adenovirus, vaccinia virus, alphavirus and reovirus - are currently active (NCT04665362, NCT04787003, NCT04386967, NCT03866525, NCT04616443, NCT03605719, NCT03206073, NCT04735978, NCT04445844, NCT03004183, NCT04755543, NCT03767348, NCT04348916, NCT04725331). For the oncolytic measles virus MV-NIS used in this study, a trial testing the combination with PD-1/PD-L1 blockade with atezolizumab in small cell lung cancer was initiated, but terminated due to low recruitment (NCT02919449).

Regarding immunological correlates, response to T-VEC plus pembrolizumab was associated with increased intratumoral CD8+ T cell infiltration, PD-L1 and IFN-γ expression after treatment (45). Based on the finding that reovirus treatment induces PD-L1 expression, a Phase I trial of intravenous reovirus and pembrolizumab in addition to chemotherapy with eleven patients suffering from pancreatic cancer was initiated. An increase in intratumoral CD8+ T cells as well as an expansion of peripheral T cell clones and increased levels of CXCL9, CXCL10 and CXCL11 in peripheral blood were observed after treatment (46).

In this study, we found increased PD-L1 expression on two of five patient-derived PDAC cultures after treatment with MV-NIS, a measles virotherapeutic currently under clinical investigation (12). This may indicate that measles virotherapy sensitizes PDAC to PD-1/PD-L1 checkpoint blockade.

Patient-derived PDAC cultures showed modest viral replication and oncolysis upon treatment with MV-NIS, indicating that direct virus-mediated cell death is limited in PDAC. Despite limited viral replication and direct virus-induced cell death also in murine cells, we found that combination treatment with MV-NIS significantly prolonged survival in a KPC (14)-derived PDAC model compared to anti-PD-1 monotherapy. This aggressive model is inherently difficult to treat and considered immunologically **“**cold**”**, reproducing key features of the tumor immune environment of human PDAC (47). For example, this model proved only transiently responsive to radio-immunotherapy (48) and refractory to combined TLR agonist and anti-PD-1 treatment (49). We have confirmed efficacy of combined measles virotherapy (Schwarz vaccine strain) and systemic PD-1 blockade in a second immunocompetent mouse model, MC38cea (10). These findings of immunotherapeutic efficacy in tumors with limited virus permissiveness add to the debate on the relevance of direct oncolysis versus *in situ* vaccination effects in immuno-virotherapy (50, 51).

Searching for correlates of immunotherapeutic efficacy, we did not detect striking alterations in PD-L1 expression, tumor-infiltrating lymphocytes, or T cell receptor repertoire upon combination immunotherapy. Rather, gene expression analysis revealed a complex immune response pattern, including activation of innate and adaptive immunity, cytokine and chemokine expression, cell adhesion, antigen presentation as well as TCR signaling. Most prominently, genes associated with Th1 polarization and activation of cytotoxic T cells were upregulated.

Our analysis initially focused on T cells, as these have been considered main targets for PD-1/PD-L1 checkpoint blockade (52). However, additional immune subsets including NK cells, dendritic cells, B cells, and macrophages have been implicated in efficacy of anti-PD-1/PD-L1 and resulting T cell responses (29, 53–59). Moreover, T cell responses and T cells reactive against high quality neoantigens resembling microbial antigens have been associated with prolonged survival in PDAC (60).

However, our results including intratumoral immune gene expression indicate that a more holistic view of the tumor immune environment is necessary to understand underlying mechanisms of response and relapse. A recent immunophenotyping study with single-cell data highlights the complexity and heterogeneity of the immune infiltrate in PDAC (61). This study identified Th1 cell abundance and the ratio of CD8+ T cells to CD168+ monocytes/macrophages as a predictor of outcome in PDAC. Thus, further investigation of the myeloid compartment in the context of immunovirotherapy may be warranted. In the KPC model, T cell immunity and STAT1 signaling have been found to trigger myeloid cells (62), which have been shown to be a major contributor to PDAC-associated immunosuppression (63). However, our gene expression data do not point towards an increase in myeloid cells at t2 as a mechanism of resistance.

Rather, a global loss of immune activation seemed to occur at t2. This may indicate activation-induced immune cell death after strong immunostimulation or reflect overriding immunosuppression in PDAC, orchestrated by malignant cell signaling, stromal barriers, and metabolic factors (63, 64). Therefore, advanced combination treatments including, e.g. CD40 agonists, TGFβ blockade, or therapeutics targeting KRAS signaling, the myeloid compartment, or tumor stroma (63, 64) may improve efficacy of virotherapy plus checkpoint blockade in PDAC.

While loss of intratumoral immune activation was observed, we detected induction of systemic anti-tumor immunity after MV therapy, which was maintained upon tumor progression. These results indicate that systemic anti-tumor immunity was driven by virotherapy and combination with anti-PD-1 mediated transient peripheral immunomodulation within the tumor. This is in line with the canonical concept of PD-1 checkpoint signaling limiting peripheral T cell responses (65). Our results indicate that additional factors restrict peripheral immune-mediated tumor clearance upon tumor progression, which must be overcome to achieve durable efficacy.

A recent study reported benefits of CAR T cell therapy combined with an oncolytic adenovirus and a helper-dependent adenoviral vector encoding IL-12 and a PD-L1 blocking minibody against pancreatic cancer xenografts (66). We have previously developed oncolytic measles viruses encoding IL-12, IL-15 agonists, PD-1 or PD-L1 blocking antibodies, and bispecific T cell engagers (6, 8, 10, 15). Thus, introducing additional immunomodulatory genes into the oncolytic vector for local expression within the tumor may be one avenue to improve outcome which avoids the risk of added toxicities.

Further considerations for improvement regard dosing and scheduling. Additional doses of virotherapy could augment efficacy. However, single administration of MV-NIS has shown clinical efficacy (67) and although debatable (68), anti-viral immunity may hamper efficacy upon repeated administration even after intratumoral injection (69). In this regard, heterologous prime-boost regimens with distinct viruses may potentiate local immunomodulation and promote durable efficacy (70). Likewise, switch and maintenance schedules for immune checkpoint blockade are currently discussed and tested in clinical trials (71). An important recent development in this regard is the implementation of PD-1/PD-L1 checkpoint blockade in a neoadjuvant setting (72). Pre-surgery anti-PD-1 induced high rates of major pathological response associated with expansion and persistance of neoantigen-specific T cells in advanced non-small cell lung cancer (73). In a randomized trial in glioblastoma, neoadjuvant anti-PD-1 was superior to adjuvant administration, with signs of local and systemic T cell activation in correlative analyses (74).

The concept of neoadjuvant immunotherapy has been extended to oncolytic virotherapy and virotherapy combined with immune checkpoint blockade (36, 40) (NCT03259425). Pre-surgical combined virotherapy and checkpoint blockade may allow for enhanced *in situ* vaccination and protect patients from relapse by residual disease or micrometases after potentially curative resection. Thus, a neoadjuvant approach may represent a more suitable setting for combination immunovirotherapy.

Limitations of our study include the restricted transferability of mouse studies to the clinical setting as well as possible sampling bias. Although the KPC model reflects the biology of human PDAC to considerable extent (47), our data show that patient-derived PDAC cultures are somewhat more permissive to MV than a KPC-derived tumor cell line. Thus, clinical efficacy against human PDAC may be enhanced compared to our preclinical model. Furthermore, as in a clinical correlative research program with distinct biopsy timepoints and scarce material, the tumor samples collected in this study may not cover all changes in the tumor microenvironment after treatment over time. Nevertheless, the signs of efficacy – though transient – and the induction of durable anti-tumor immunity in this model indicate that combined measles virotherapy and PD-1 checkpoint blockade warrants further investigation in PDAC.

## Data availability statement

The original contributions presented in the study are included in the article/**Supplementary Material**. Further inquiries can be directed to the corresponding author.

## Ethics statement

The studies involving human participants were reviewed and approved by Ethics committee of the Medical Faculty of Heidelberg University (323/2004, Amendment 03). The patients/participants provided their written informed consent to participate in this study. The animal study was reviewed and approved by Regierungspräsidium Karlsruhe, protocols G-192/15, G-58/17, G-17/19.

## Author contributions

Conceptualization: CE, RV. Methodology: RV, GP-M, CB, LK. Investigation and analyses: RV, GP-M, CM, CT, TS, JL, LK. Writing – original draft: CE, RV, GP-M.; Writing – review and editing: all authors. Resources: GU, AS and DJ. All authors contributed to the article and approved the submitted version.
