# Supplementary material for: Virotherapy combined with anti-PD-1 transiently reshapes the tumor immune environment and induces anti-tumor immunity in a preclinical PDAC model
Source: Front Immunol. 2023 Jan 16;13:1096162. doi: 10.3389/fimmu.2022.1096162 (PMC9886093; doi:10.3389/fimmu.2022.1096162)
Supplement: Supplementary file 1 [file DataSheet_1.pdf]

Figure S1

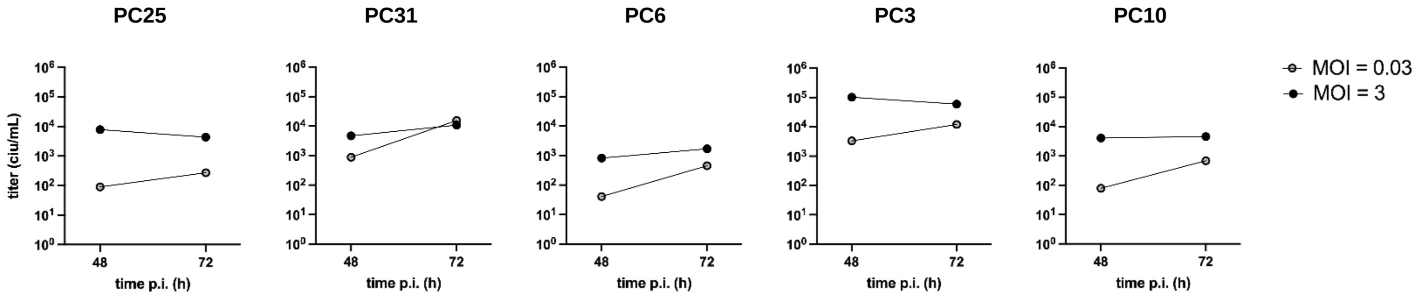

Supplementary Figure S1. MV-NIS replication in patient-derived PDAC cultures. PDAC cultures were infected with MV-NIS at MOI = 0.03 and MOI = 3. Viral progeny at 48 and 72 h post infection (p.i.) were determined by titration assays.

Figure S2

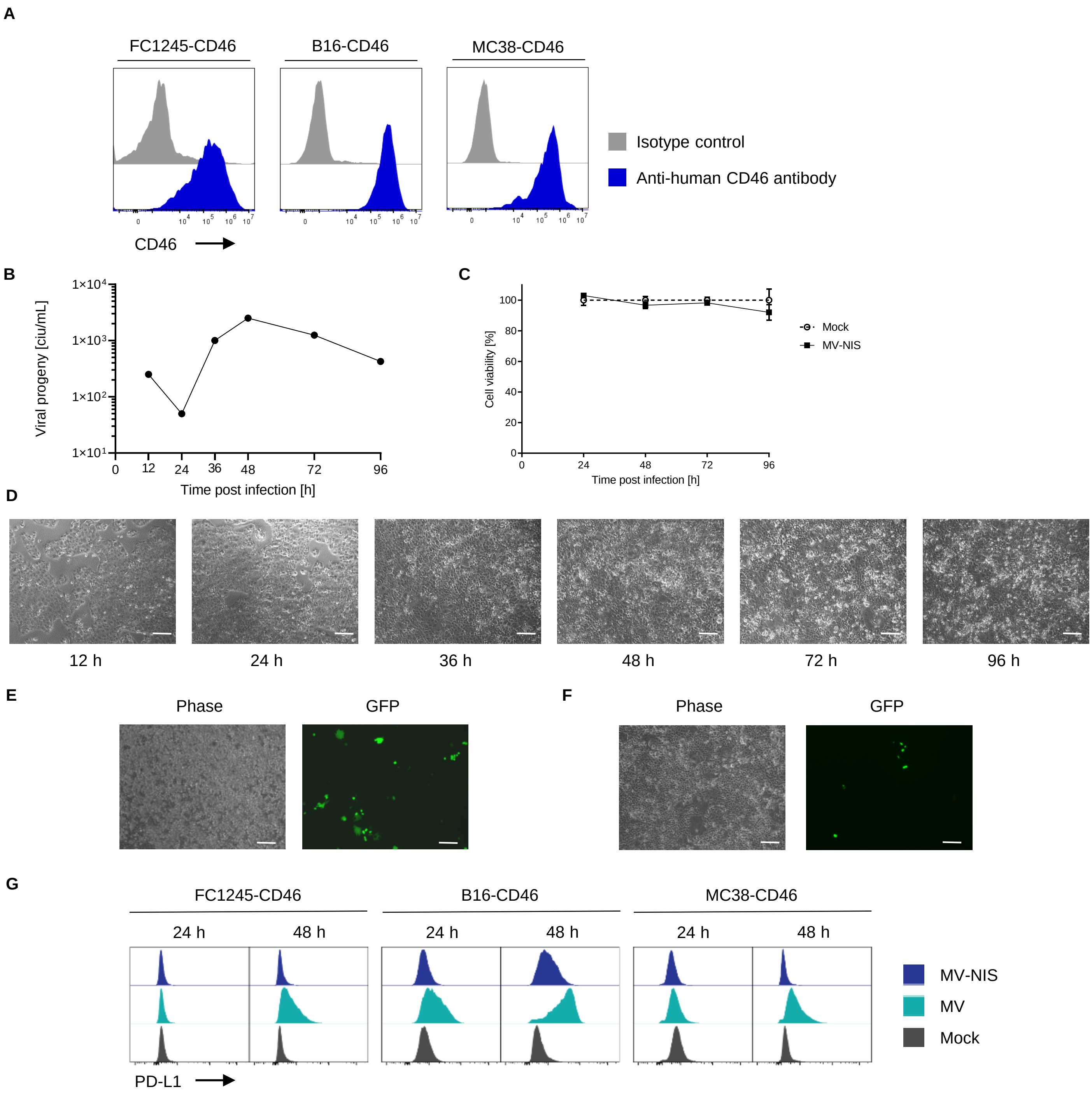

Supplementary Figure S2. MV susceptibility and PD-L1 expression in murine tumor cell lines. A Expression of the MV entry receptor CD46 on engineered murine tumor cell lines was determined by flow cytometry. **B - D** FC1245-CD46 cells derived from the KPC model of PDAC were infected with MV-NIS at MOI = 3 or subjected to mock infection. **B** One-step growth curve analysis. At designated timepoints post infection, cells and cell culture supernatants were harvested by scraping cells in cell culture media. Viral progeny in cell infectious units per ml (ciu/ml) were determined by serial dilution titration assay. **C** Cell viability assay. At designated timepoints after infection with MV-NIS, cell viability was assessed by XTT assay. Viability of mock infected cells was defined as 100%. Mean values of triplicate infections are shown. Error bars indicate standard deviation. **D** Microscopy images corresponding to timepoints depicted in B. **E** FC1245-CD46 cells were infected with a measles vaccine variant encoding eGFP at MOI 1. Images were acquired 72 h post infection. **F** FC1245-CD46 cells were injected subcutaneously into the flanks of C57BL/6 mice. After two weeks, tumors were explanted and cultivated ex vivo until confluent (two weeks). Cultures were infected with a measles vaccine variant encoding eGFP at MOI 1. Images were acquired 72 h post infection. **G** FC1245-CD46, B16-CD46 and MC38-CD46 cells were infected with MV-NIS or measles Schwarz vaccine strain (MV) or subjected to mock infection. Twenty-four and 48 hours post infection, PD-L1 expression was assessed by flow cytometry. D, E, F: Scale bars: 100 µm.

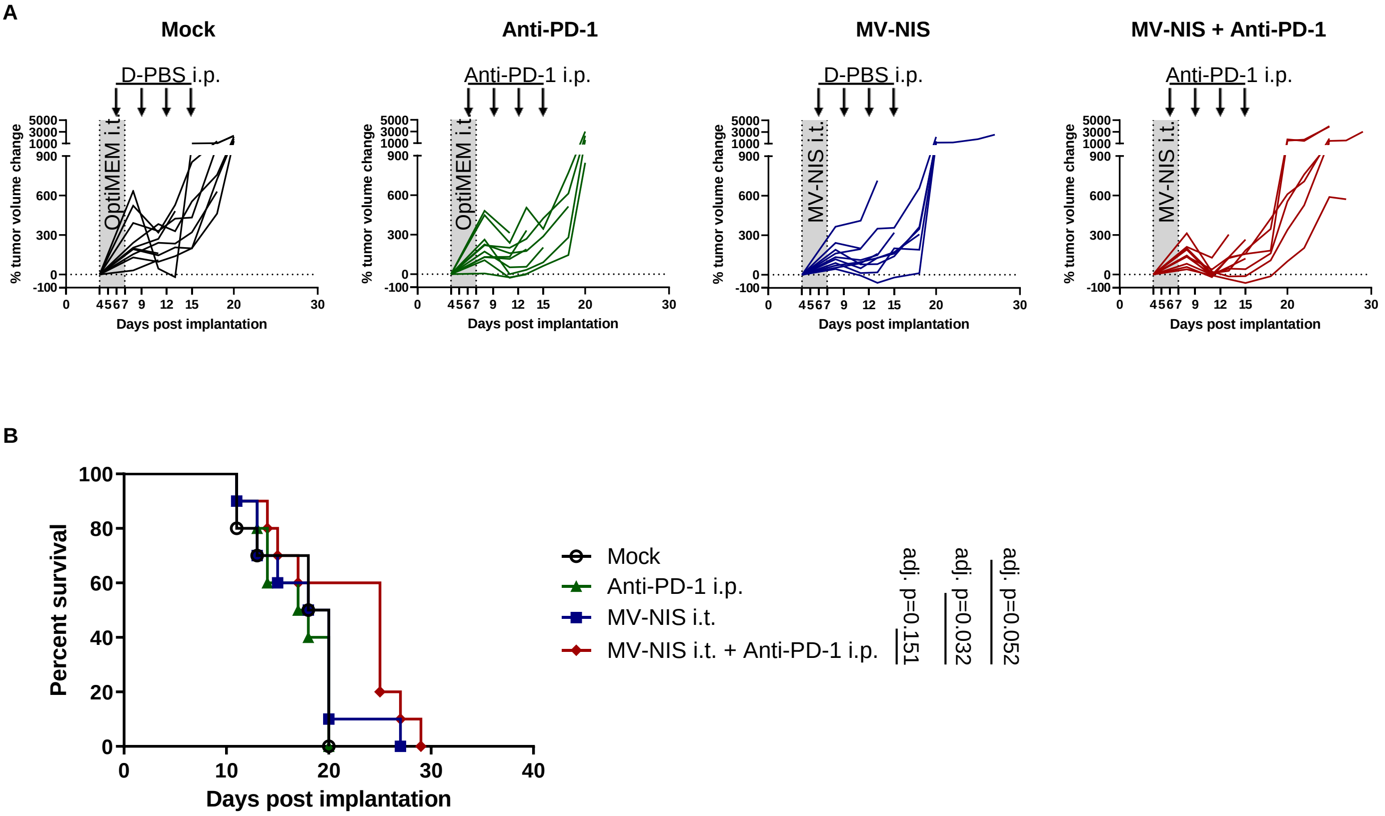

Supplementary Figure S3. Repetition of experiment shown in Figure 2. **A** Individual tumor growth curves. Relative changes in tumor volume in comparison to pre-treatment tumor volume over time are shown. **B** Kaplan-Meier survival analysis. Data was analyzed using Mantel-Cox (log rank) test with Bonferroni correction for multiple comparisons.

Figure S4

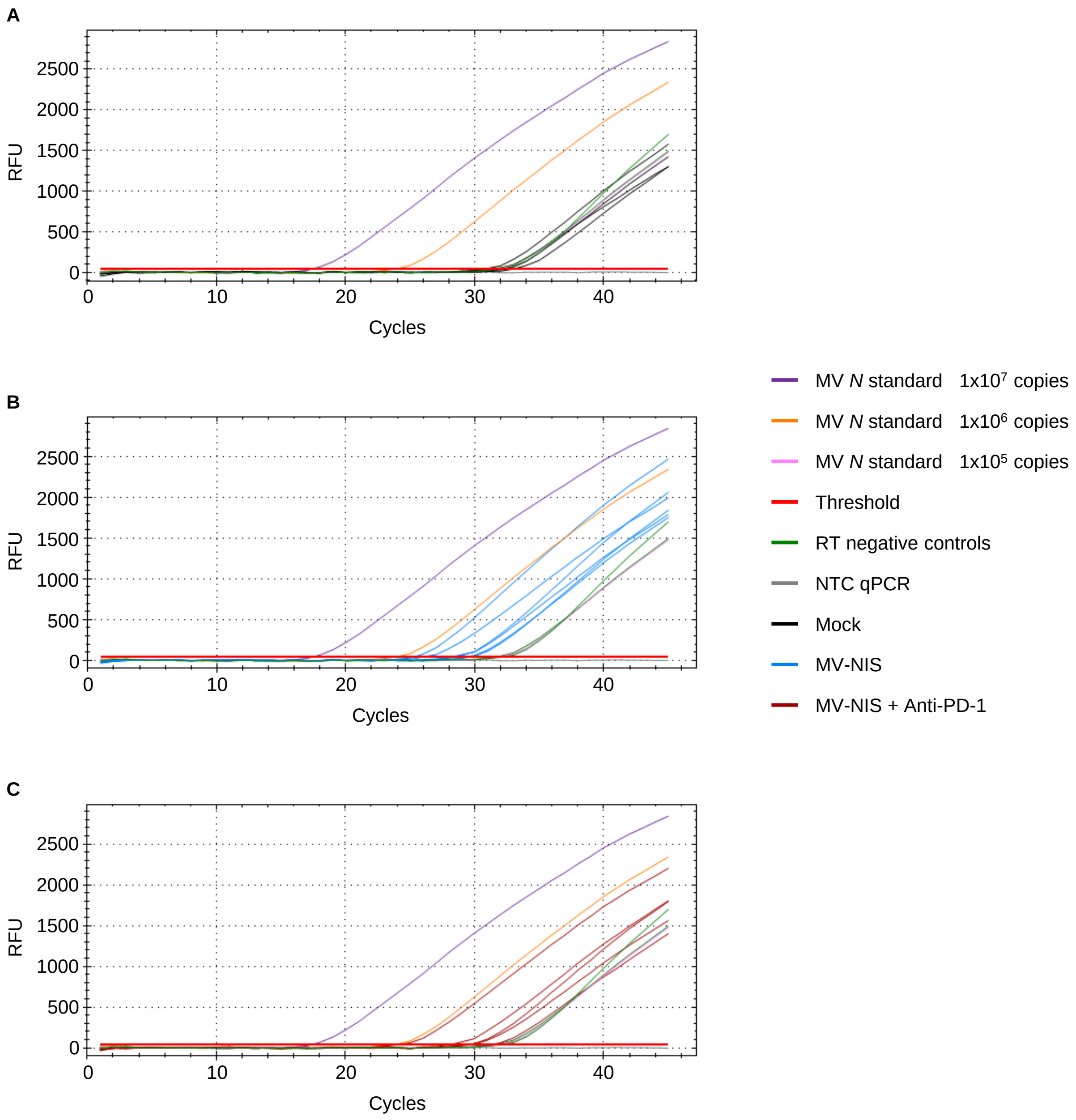

Supplementary Figure S4. Detection of intratumoral MV *N*. Tumors explanted at t1 as depicted in Figure 3 were processed for qPCR analysis of MV *N* gene. Amplification curves are shown for tumor samples after **(A)** mock, **(B)** MV-NIS, and **(C)** MV-NIS + Anti-PD-1 treatment. Amplification curves are shown for MV *N* standards, reverse transcription (RT) negative controls and qPCR non-template control (NTC). Red horizontal line indicates threshold.

Figure S5

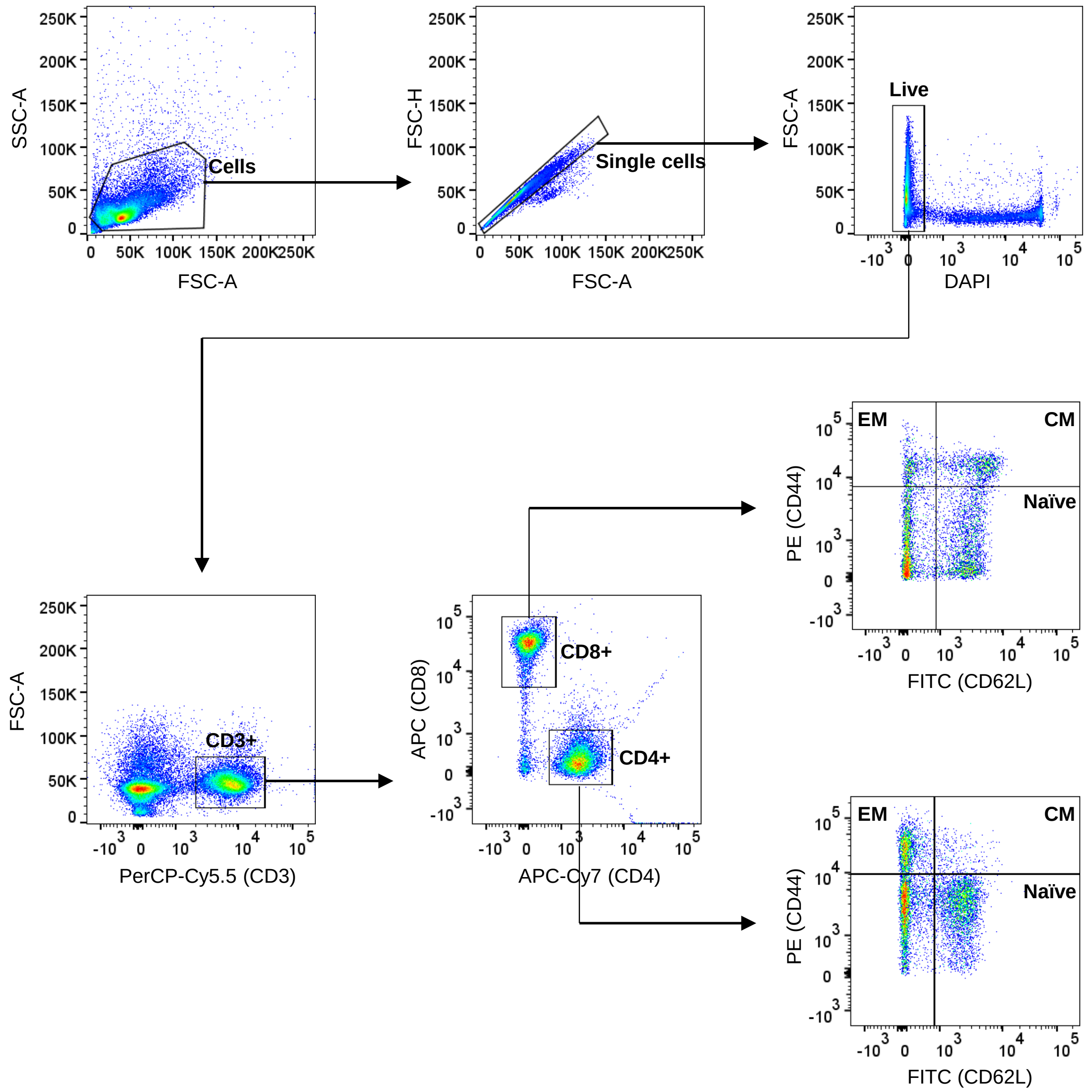

Supplementary Figure S5. Gating strategy for flow cytometry of tumor-draining lymph nodes. EM: effector memory T cells. CM: central memory T cells.

Figure S6

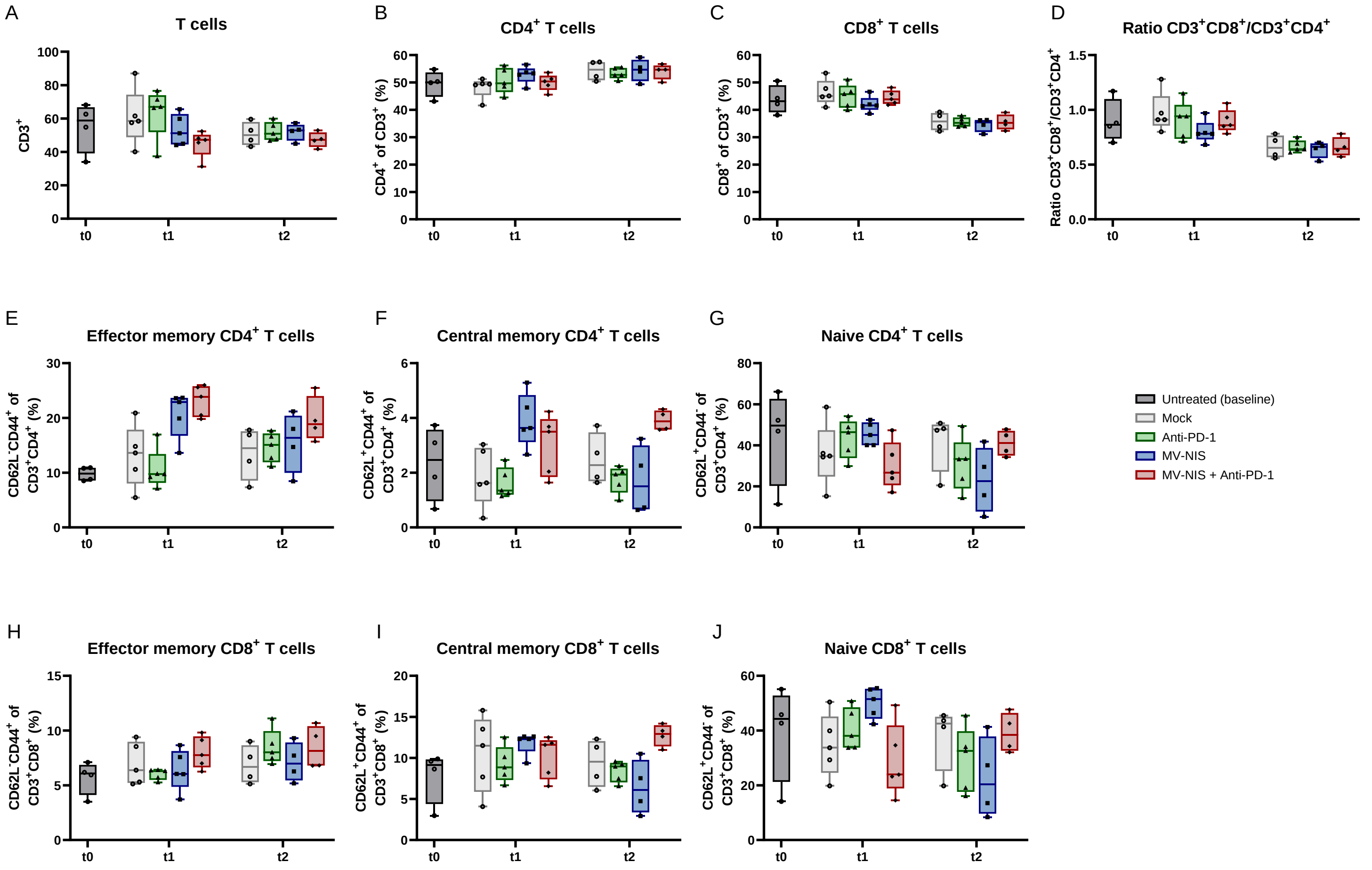

Supplementary Figure S6. Flow cytometry of tumor-draining lymph nodes.
Dots representing individual tumors and median in the group are shown. The corresponding gating strategy is depicted in Figure S5. Box and whisker plots with whiskers depicting minimal and maximal detected abundances as well as median in each group are shown. Dots represent individual tumor samples. Data were analyzed by one-way ANOVA for each timepoint with Tukey's post-test. Multiplicity-adjusted p values <0.05 are shown. adj. p = adjusted p value.

Figure S7

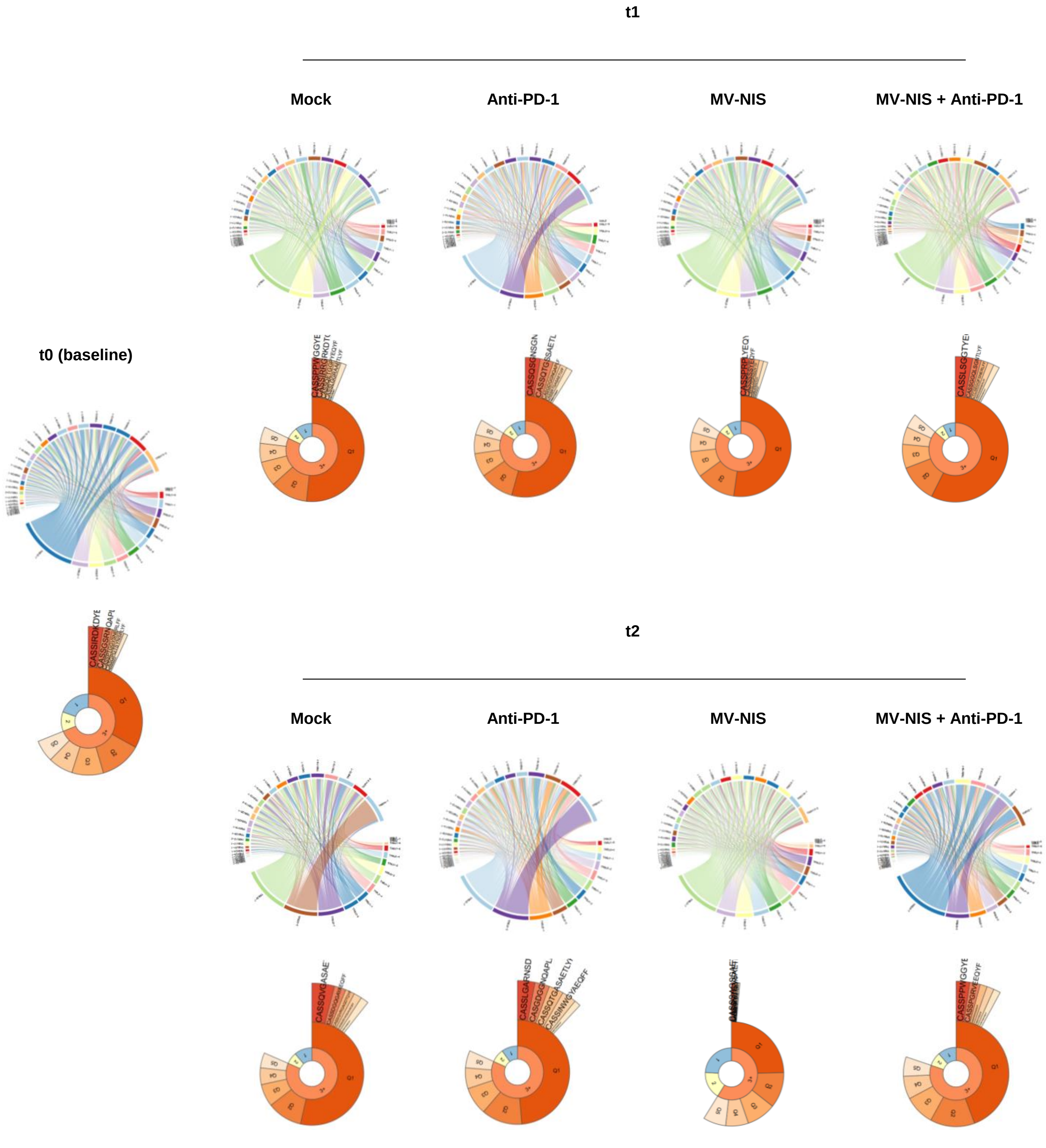

Supplementary Figure S7. TCR analysis- VJ usage and quantile statistics. Pooled data from four mice in each group at each timepoint based on strict intersection rule were analyzed using vdjtools (21). VJ segment usage (circos plots) and quantile statistics (donut plots) are depicted. In the latter, the first layer includes the frequency of singleton ("1", met once), doubleton ("2", met twice) and high-order ("3+", met three or more times) clonotypes. The second layer ("quantile"), displays the abundance of top 20% ("Q1"), next 20% ("Q2"), … (up to "Q5") clonotypes for clonotypes from "3+" set. The last layer ("top") displays the individual abundances of top N clonotypes.

Figure S8

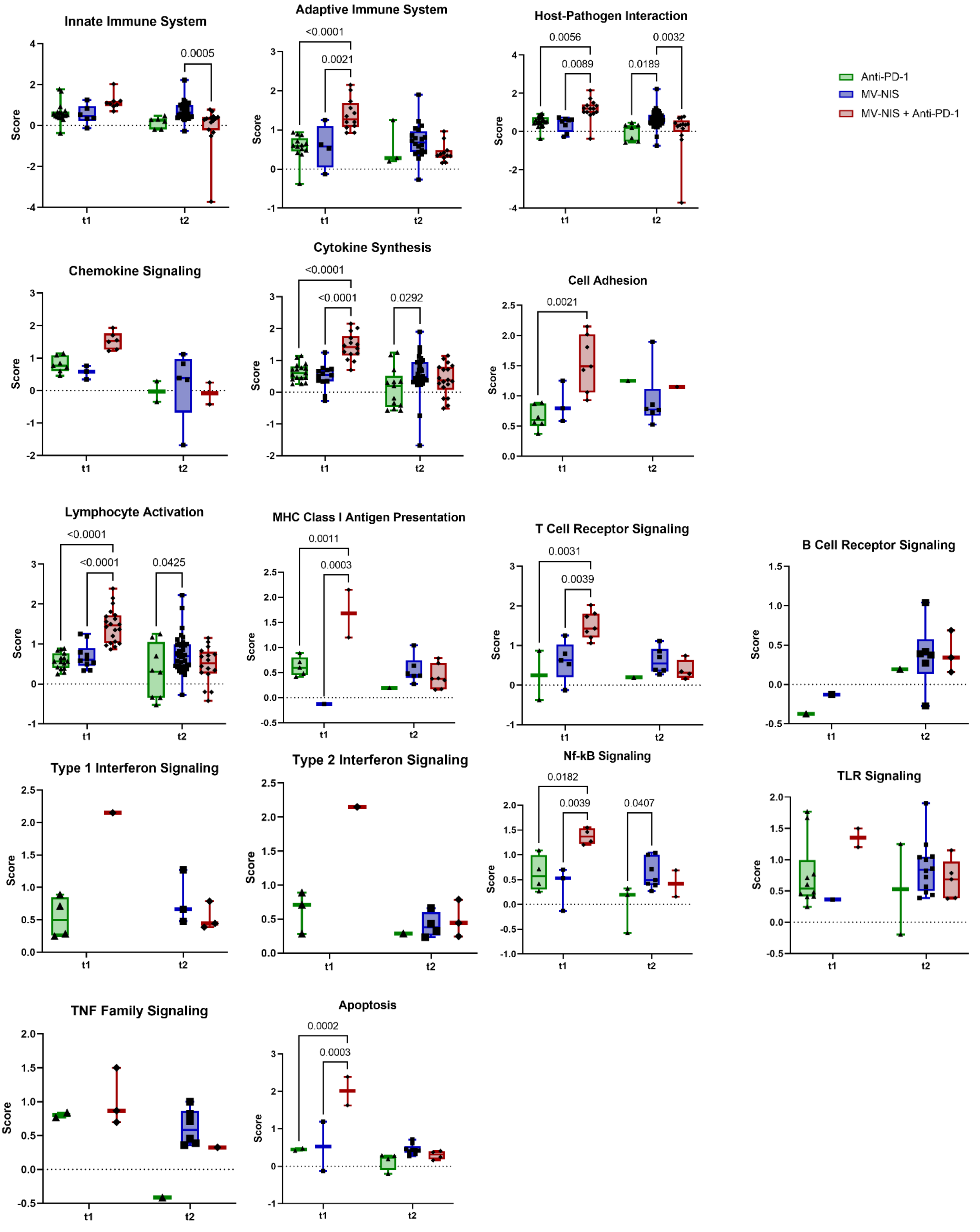

Supplementary Figure S8. Pathway analysis. Nanostring gene expression profiling data were analyzed with nSolver™ software using the Advanced Analysis package. Genes differentially expressed between mock and treatment groups ($p < 0.05$) were mapped onto pathways using nSolver™ software. log2-fold change expression values for each differentially expressed gene are shown. Box and whisker plots with whiskers depicting minimal and maximal detected abundances as well as median in each group are shown. For pathways with data for all groups at t1 and t2, two-way ANOVA with Tukey's post-test was performed. Adjusted p values for $p < 0.05$ are shown.

Figure S9

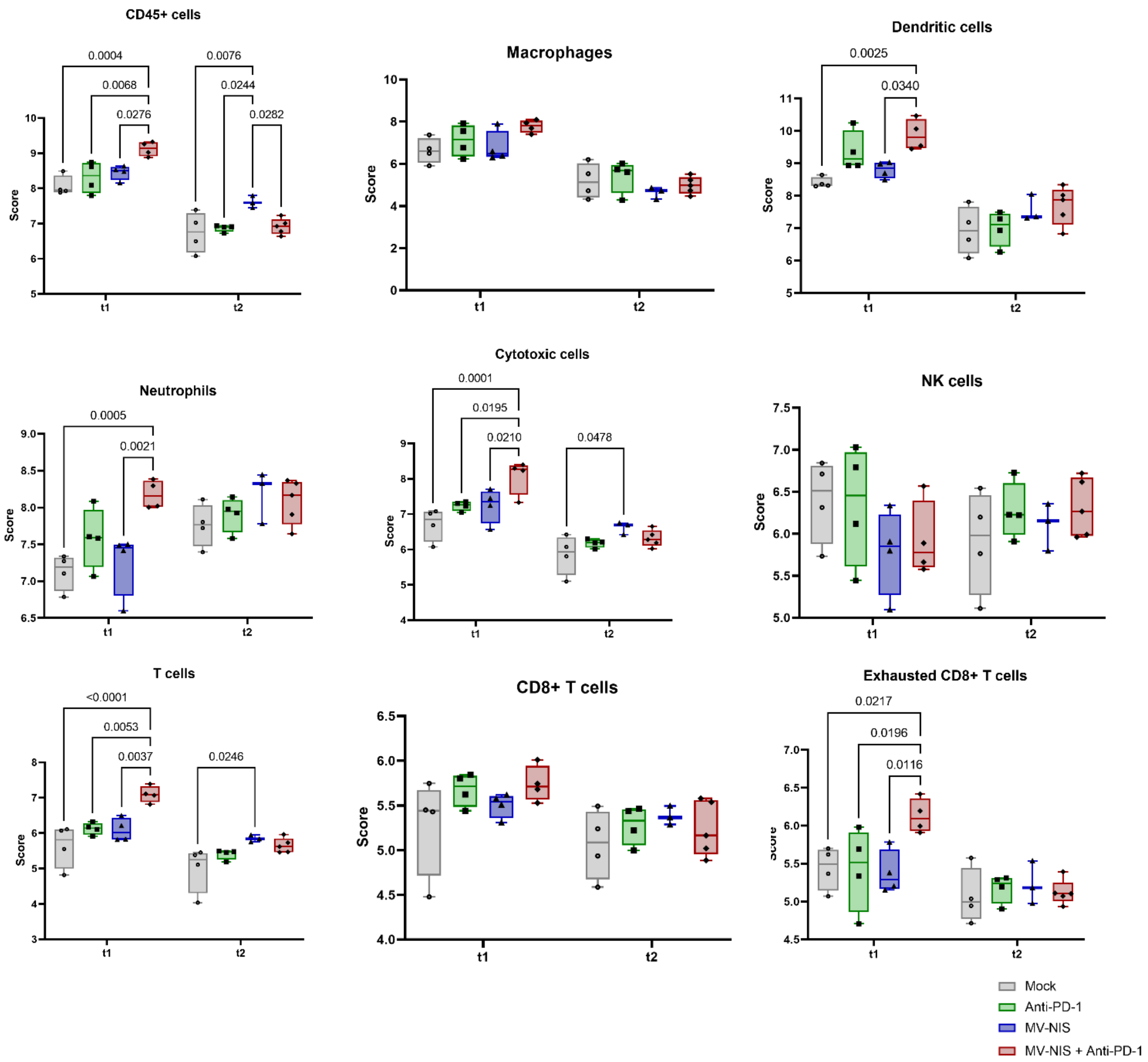

Supplementary Figure S9. Immune cell deconvolution. Nanostring data was used for immune cell deconvolution with the Advanced Analysis package of nSolver™ software. Cell type profiling scores are shown. Box and whisker plots with whiskers depicting minimal and maximal scores as well as median in each group are shown. Dots represent individual tumor samples. Two-way ANOVA with Tukey's post-test was performed. Adjusted p values for $p < 0.05$ are shown.

Figure S10

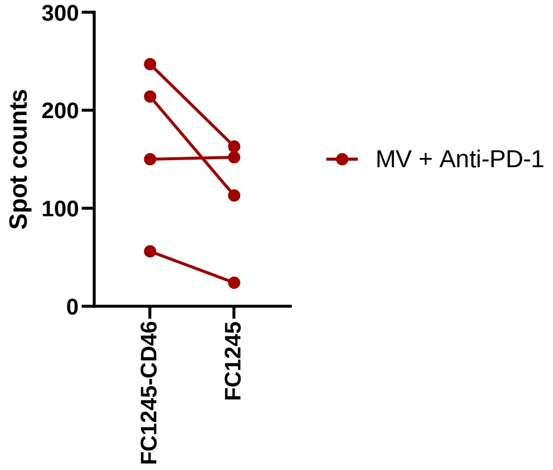

Supplementary Figure S10. Systemic anti-tumor immunity after MV plus anti-PD-1 treatment. C57BL/6J mice bearing subcutaneous FC1245-CD46 tumors were treated with MV and anti-PD-1 as described in methods, with treatment starting 5 days after tumor cell implantation. Spleens were collected at t1. $1x10^6$ splenocytes were cultured with $1x10^5$ FC1245-CD46 or parental FC1245 cells at a 10:1 effector to target ratio and IFN-γ ELISpot was conducted. Spot counts for FC1245-CD46 and parental FC1245 cells are shown with lines connecting results from individual mice.
